# Patients’ perspectives on palliative chemotherapy of colorectal and non - colorectal cancer: a prospective study in a chemotherapy- experienced population

**DOI:** 10.1186/1471-2407-13-66

**Published:** 2013-02-07

**Authors:** Marika Mende, Karolin Trautmann, Anke Rentsch, Beate Hornemann, Ulrich S Schuler, Gerhard Ehninger, Gunnar Folprecht

**Affiliations:** 1 Medical Department I, University Hospital Carl Gustav Carus, Fetscherstraße 74, 01307, Dresden, Germany; 2 University Cancer Center, University Hospital Carl Gustav Carus, Fetscherstraße 74, 01307, Dresden, Germany; 3 Department of Psychooncology, University Hospital Carl Gustav Carus, Fetscherstraße 74, 01307, Dresden, Germany; 4 Department of Palliative Care, University Hospital Carl Gustav Carus, Fetscherstraße 74, 01307, Dresden, Germany

**Keywords:** Chemotherapy, Palliative care, Survival threshold, Fatigue

## Abstract

**Background:**

A better understanding of patients’ views on the benefit and burden obtained from palliative chemotherapy would facilitate shared decision making. We evaluated palliative cancer patients’ reported outcomes (PROs) for toxicity and investigated the survival threshold for which they would repeat chemotherapy (CTx).

**Methods:**

Patients who had received a minimum of three months of palliative CTx for advanced colorectal (CRC) or non-colorectal (non-CRC: upper gastrointestinal, lung and head-and-neck) cancer were assessed by questionnaire. Patients were questioned about PROs for toxicity, subjective burden from side effects, and were asked for the survival threshold necessary for them to repeat CTx. Expected survival (sum of indicated survival threshold and median survival time with best supportive care) was compared to the patients’ actual survival.

**Results:**

One hundred and thirty-four patients (CRC: 58; non-CRC: 76) were surveyed. The most frequent PRO- grade 3/4 toxicities were acne (12.8%), fatigue (9.0%), and diarrhea (8.5%). The symptom causing the highest subjective burden was fatigue and was worse than expected in 29.9% of the patients. The median survival threshold for which patients would repeat CTx was significantly longer in CRC than in non-CRC patients (*p*=0.01). Median expected survival was significantly longer than actual median survival (CRC: 44.0 months [22.0-65.9] compared with 30.0 months of actual survival [20.9-39.1]; non-CRC: 22.0 months [15.3-28.6] compared with 19.0 months of actual survival [15.1-22.9], *p*=0.03).

**Conclusion:**

Fatigue deserves more attention when toxicity of treatment and symptoms of disease are explained to patients. Patients’ survival expectations from palliative chemotherapy are higher than previously described, exceed the median survival time known from phase III trials, and are significantly longer than their actual survival.

## Background

Cancer rates are increasing worldwide with a predicted incidence of 15 million cases in 2015 [1]. Many cancer patients are faced with incurable disease, requiring palliative therapy. In recent years, there has been a significant improvement in overall survival with palliative chemo- and antibody-based therapies in various malignancies, especially colorectal cancer [2-5]. In most clinical studies evaluating new cancer therapies, objective efficacy parameters like overall and progression-free survival as well as safety parameters are typical primary endpoints. In contrast, so-called “PROs” (patient-reported outcomes), defining subjective measures with the focus on patients’ perspectives are less frequently investigated [6]. Only a few studies have explored patients’ attitudes toward therapy, particularly in relation to survival benefit from chemotherapy [7-14]. In the main, these studies indicated that cancer patients accept toxic therapies for smaller survival benefits than do healthy control groups, consisting of doctors, nurses and volunteers [7-11]. As communication in palliative cancer care is challenging, a better understanding of patients’ views on palliative chemotherapy would help to facilitate shared decision making in such difficult treatment situations [15]. Here, we evaluated the subjective impact of common chemotherapy-related side effects. We aimed to find out the survival threshold at which palliative cancer patients would consider chemotherapy worthwhile and identified potential factors influencing the extent of the indicated survival threshold. Patients’ survival expectations were compared to their actual survival times.

## Methods

### Patients

The survey was conducted at our institution from August 2008 until December 2009. Patients who had completed a minimum of three months of palliative chemotherapy for colorectal cancer (CRC), cancer of the upper gastrointestinal tract (u-GI), non-small cell lung cancer (NSCLC), and squamous cell cancer of the head and neck (SCCHN) were eligible. Patients were eligible independent of prior cancer therapies including surgery, adjuvant chemo-, or radiation therapy. The standard patient informed consent for chemotherapy at the institution included an emphasised section describing the palliative aim of therapy.

Analysis was planned for two disease groups reflecting their different prognoses: CRC versus non-CRC (u-GI, NSCLC and SCCHN). Patients’ demographic and treatment data, including age, gender, start of first palliative therapy, current and prior regime as well as date of death were acquired from the database of the Dresden University Cancer Center. Treating physicians and nurses were blinded to the results. All patients had given their informed consent before inclusion into the study. The ethics committee of the University of Dresden approved the study.

### Questionnaire

The questionnaire was developed for the current study. The questionnaire evaluated common chemotherapy-related toxicities including stomatitis, nausea, vomiting, diarrhea, fatigue, sensory neuropathy, acne, and alopecia according to NCI-CT criteria [16] as well as pain [17]. Patients indicated the subjective burden from reported toxicity on a numeric rating scale with a scale from 0 to 10. Choosing the number 0 would mean “no symptom” and 10 would mean “worst possible symptom”. The interpretation of the rating scales followed the interpretation of the pain scale as described by Serlin (1-4 mild, 5-6 moderate, 7-10 severe) [17]. Additionally, patients were asked to recall the severity of any toxicity compared with their original expectations after informed consent. For this purpose they could choose between five different scenarios according to a five-point Likert scale (“much less than expected, less than expected, just as expected, more than expected, much more than expected”) [18]. Patients were asked whether they would repeat therapy. Possible answers included “probably yes, yes, unsure, probably no, and no”. Finally, patients were requested to state the survival threshold in months necessary for them to repeat therapy. The “hospital anxiety and depression scale” (HADS), a common screening method for anxiety disorders (HADS-A) and depression (HADS-D) in physically ill people was used to assess psychological morbidity [19,20]. The questionnaire was handed out by a cancer nurse at a regular patient appointment and had to be returned in an enclosed envelope. The original questionnaire can be found in the Additional file 1: Figure S4.

### Analysis

The following two variables must be defined before further explanation of analysis of the current descriptive study: *Survival threshold* is the minimum survival benefit in treatment. *Expected survival* is the sum of survival threshold as stated by the patients and median survival with best supportive care as reported in the literature. Studies suggest a median survival with best supportive care of eight months for CRC patients and of four months for non-CRC patients [21-24].

Data were analyzed using SPSS version 17.0. Frequency counts were conducted for descriptive analysis. Bar charts summarize the frequency of chemotherapy-related, patient-reported toxicities and the subjective burden from toxicity as well as the extent of occurrence of adverse events in comparison with expectation after informed consent. Kaplan-Meier survival analysis was performed to compare actual survival to expected survival for CRC and non-CRC patients. To determine the influence of disease group, toxicity, pain, and psychological distress on the extent of the anticipated survival threshold, multi-factorial ANOVA was done. P-values less than 0.05 were considered statistically significant.

## Results

**Figure 1:**
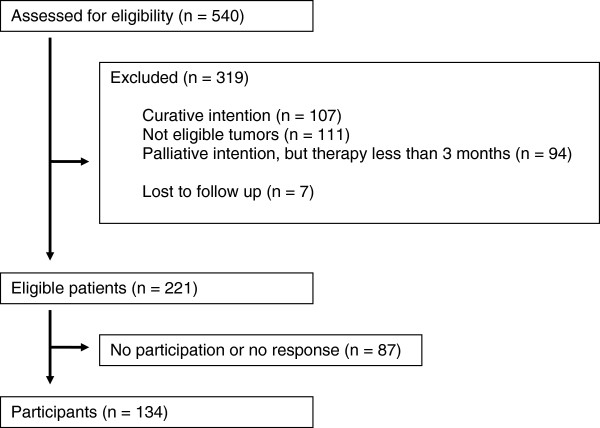
**Flow diagram. **Inclusion in the study.

Between August 1^st^, 2008 and December 31^st^, 2009, 540 consecutive patients receiving chemotherapy at our institution were screened for the study as shown in Figure 1. Two hundred and twenty-one patients were eligible but 87 patients did not participate or failed to respond. Of the 134 included patients, 58 patients suffered from CRC, 76 from non-CRC (u-GI: 45, NSCLC: 18, SCCHN: 13). The median age was 63 years (range 32–86 years); 71.0% of the patients were male. Patients had completed a median of six months (range 3-51 months) of palliative chemotherapy before entering the study. One hundred and six patients (79.0%) had died by the end of follow-up in July 2011. Patients’ characteristics are summarized in Table 1. CRC patients were mainly treated with irinotecan- or platinum- based therapies. Patients in the non-CRC group received platinum- or gemcitabine-based therapies. In both groups, therapy was usually a combination with a second cytotoxic drug and/or a monoclonal antibody. Platinum-containing therapy was more common in non-CRC patients (54.0%) than in CRC patients (32.8%) at the time of study (*p*=0.02). Ten patients in the non-CRC group were only treated with tyrosine kinase inhibitors at the time of the survey but had previously received at least three months of conventional chemotherapy. For more information about chemotherapy at the time of study and prior to study see Additional file 1: Table S1B in the Additional file 1.

**Table 1:** Patients’ characteristics

**Patients’ characteristics**	**Entity**					
	**CRC n=58**	**Non-CRC n=76**	**u-GI n=45**	**NSCLC n=18**	**SCCHN n=13**	**all n=134**
**Age**						
Median (Range)	63 (32-78)	61 (33-86)				62 (32-86)
IQR	58-68	54-67				55-68
< 60 yrs	20 (34.0%)	35 (46.0%)	18	9	8	55 (41.0%)
60-69 yrs	28 (48.0%)	27 (36.0%)	21	3	3	55 (41.0%)
≥ 70 yrs	10 (17.0%)	14 (18.0%)	6	6	2	24 (18.0%)
**Sex**						
Male	45 (78.0%)	50 (70.0%)	28	12	10	95 (71.0%)
Female	13 (22.0%)	26 (17.0%)	17	6	3	39 (29.0%)
**Psychological** Morbidity *						
Anxiety	1 (2.0%)	14 (18.0%)	8	2	4	15 (11.0%)
Depression	3 (5.0%)	13 (17.0%)	8	3	2	16 (12.0%)
Median time of CTx	8	4	4	7	3,5	6
at survey (months)°						
Number of deaths	44 (76.0%)	62 (82.0%)	36	14	12	106 (79.0%)

* Psychological morbidities (anxiety disorder, depression) measured by HADS, 131 responding patients.

° Median time of CTx at survey in months.

### Toxicity

**Figure 2:**
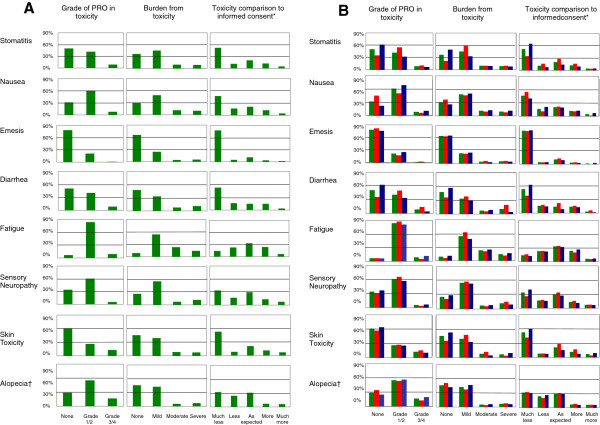
**PRO in toxicity for all patients. **Green: All Patients. * Patients’ expectations in comparison to informed consent: much less, less, more, and much more than expected. † Grades of patient-reported toxicity for alopecia are: none, grade 1, grade 2.

A synopsis of patient-reported toxicity is shown in Figure 2 and Table 2 as well as in Additional file 1: Figure S2B. The most common toxicity of any PRO-grade was fatigue (93.2%). Overall, the most frequently reported grade 3/4 toxicity was acne (12.8%), followed by fatigue (9.0%), diarrhea (8.5%), stomatitis (8.3%), and nausea (8.1%). In CRC patients, acne was the most commonly reported grade 3/4 toxicity. In non-CRC patients, fatigue was the most common toxicity. Irrespective of disease group, patients felt severely burdened by fatigue (14.3%), sensory neuropathy (12.0%), diarrhea (11.1%), and nausea (9.8%). Whereas fatigue was the symptom with the highest impact on non-CRC patients, CRC patients felt especially burdened by diarrhea. In comparison with patients’ expectations at informed consent, fatigue occurred “more” and even “much more” than expected in 29.9% of patients. Other side effects that occurred more often than initially expected were sensory neuropathy in 20.5% of the patients and acne in 18.4% of the patients. Vomiting occurred less than expected in 83.0% of the patients.

**Table 2:** Quantitative analysis of PROs in toxicity

**Grade of PRO in toxicity**	**Entity**				
	***CRC***	***Non-CRC***	***u-GI***	***NSCLC***	***SCCHN***
***Stomatitis***	*n= 57*	*n= 75*	*n=45*	*n=18*	*n=12*
None	35.1%	61.3%	66.7%	66.7%	33.4%
Grade 1/2	54.4%	32.0%	31.1%	27.8%	41.6%
Grade 3/4	10.5%	6.7%	2.2%	5.5%	25.0%
***Nausea***	*n*=*54*	*n*=*70*	*n*=*44*	*n*=*14*	*n*=*12*
None	44.4%	21.4%	18.2%	21.5%	33.4%
Grade 1/2	50.0%	68.6%	72.8%	71.4%	50.0%
Grade 3/4	5.6%	10.0%	9.0%	7.1%	16.6%
***Emesis***	*n*=*54*	*n*=*74*	*n*=*42*	*n*=*18*	*n*=*13*
None	81.4%	75.7%	76.2%	72.3%	77.0%
Grade 1/2	16.7%	24.3%	23.8%	27.7%	23.0%
Grade 3/4	1.9%	0.0%	0.0%	0.0%	0.0%
***Diarrhea***	*n*=*56*	*n*=*74*	*n*=*44*	*n*=*17*	*n*=*13*
None	35.7%	62.1%	54.6%	76.5%	69.3%
Grade 1/2	50.0%	33.8%	38.6%	23.5%	30.7%
Grade 3/4	14.3%	4.1%	6.8%	0.0%	0.0%
***Fatigue***	*n*=*57*	*n*=*76*	*n*=*45*	*n*=*18*	*n*=*13*
None	7.0%	6.6%	6.7%	5.6%	7.7%
Grade 1/2	87.7%	81.6%	75.6%	94.4%	84.6%
Grade 3/4	5.3%	11.8%	17.7%	0.0%	7.7%
***Sens*. *Neu*.**	*n*=*55*	*n*=*74*	*n*=*44*	*n*=*17*	*n*=*13*
None	40.0%	36.5%	31.9%	41.2%	46.1%
Grade 1/2	65.4%	56.7%	59.1%	58.8%	46.1%
Grade 3/4	3.6%	6.8%	9.0%	0.0%	7.8%
***Skin toxicity***	*n*=*58*	*n*=*75*	*n*=*45*	*n*=*17*	*n*=*13*
None	57.0%	64.0%	73.4%	64.8%	30.8%
Grade 1/2	27.5%	25.4%	24.4%	11.7%	46.1%
Grade 3/4	15.5%	10.6%	2.2%	23.5%	23.0%
***Alopecia***	*n*=*56*	*n*=*73*	*n*=*45*	*n*=*16*	*n*=*12*
None	33.9%	24.6%	26.7%	18.75%	25.0%
Grade1	53.6%	56.2%	60.0%	50.0%	50.0%
Grade 2	12.5%	19.2%	13.3%	31.25%	25.0%

Sens. Neu. = Sensory Neuropathy.

### HADS

A total of 130 patients completed the HADS section of the questionnaire. Fifteen patients (11.5%) with possible anxiety disorders were identified by HADS-A (CRC: 1; non-CRC: 14; *p*=0.002). According to HADS-D, possible depression occurred in 16 patients (12.3%; CRC: 3; non-CRC: 13; *p*=0.056). Eight patients (CRC: 1; non-CRC: 7) were likely to be affected by both, anxiety disorder and depression. Patients in the non-CRC group were more likely to show abnormal scores in any subscale of HADS, compatible with psychological morbidity, than in the CRC group.

### Survival threshold

A total of 131 out of 134 patients answered the question whether they would repeat therapy. Eighty-eight percent stated “yes” or “probably yes” while 9.0% were unsure, and 3.0% responded “probably no” and “no”. Ninety-seven patients (72.3%) answered the question concerning the anticipated survival threshold. The following results refer to these patients only. Answers varied from “even a single day is worth it” (equivalent to zero months) up to the expectation of a survival threshold of 340 months. In summary, CRC patients considered a median threshold of 36 months (95% CI: 24.0 to 60.0) to be worthwhile repeating therapy for. For non-CRC patients, a median survival threshold of 18 months (95% CI: 12.0 to 24.0) would be necessary to repeat therapy. This difference was statistically significant (*p*=0.01).

### Expected vs. actual survival

**Figure 3:**
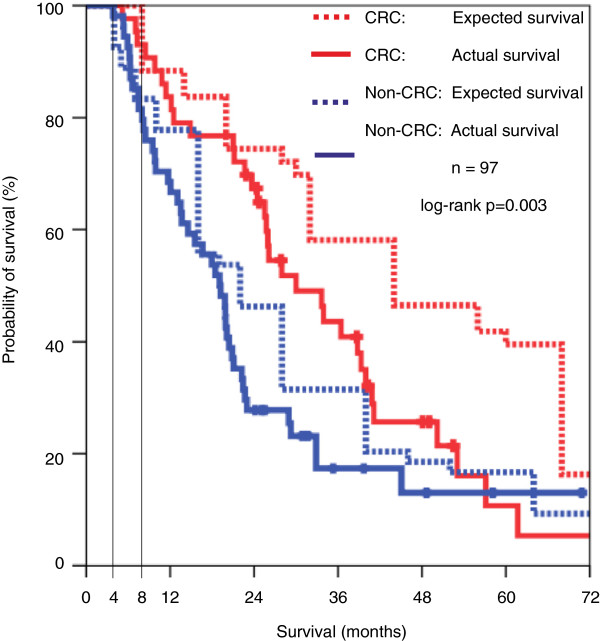
**Survival Expectation and Actual Survival. **The figure shows actual survival (solid lines) and “expected survival” (dotted lines) of patients with colorectal and non-colorectal solid tumours. Log-rank test combined for both strata: *p = *0.003. Horizontal lines mark median survival time with best supportive care known from studies. For CRC: Eight months. For Non-CRC: Four months. The patient-reported survival threshold was added to median survival time with best supportive care.

After adding the median survival time with best supportive care to the patients’ stated survival thresholds, this expected survival was plotted against the patients’ actual survival as shown in Figure 3. In the CRC group, the median expected survival was 44.0 months (95% CI: 22.0 to 65.9) compared with a median actual survival of 30.0 months (95% CI: 20.9 to 39.1). Non-CRC patients’ median expected survival was 22.0 months (95% CI: 15.3 to 28.6) compared with a median actual survival of 19.0 months (95% CI: 15.1 to 22.9). Expected survival was significantly longer than the actual survival. (*p*=0.003; adjusted for disease group).

### Independent factors for magnitude of survival benefits

Multifactorial analysis suggested that disease group, depression, and diarrhea were factors independently influencing the extent of the anticipated survival threshold. Non-CRC patients were willing to repeat therapy for smaller survival thresholds than CRC patients. Patients with higher scores in the HADS-D (equivalent to possible depression) and those with higher toxicity grades for diarrhea would repeat therapy for a lower survival threshold as well (Table 3).

**Table 3:** Multi-factorial ANOVA

**Independent factors**	**Survival threshold**	
	**Regression coefficient**	**Significance**
***Disease group***		
CRC vs. Non-CRC	- 0.289	0.032
***Sex***		
Male vs. Female	- 0.013	0.921
***Age***		
< 60 vs. 60-69 vs. ≥ 70	- 0.054	0.638
***Anxiety***		
None vs. Possible vs. Likely	0.195	0.168
***Depression***		
None vs. Possible vs. Likely	- 0.439	0.020
***Stomatitis***		
None vs. Grade 1/2 vs. Grade 3/4	0.209	0.117
***Nausea***		
None vs. Grade 1/2 vs. Grade 3/4	0.110	0.424
***Vomiting***		
None vs. Grade 1/2 vs. Grade 3/4	−0.078	0.564
***Diarrhea***		
None vs. Grade 1/2 vs. Grade 3/4	- 0.267	0.032
***Fatigue***		
None vs. Grade 1/2 vs. Grade 3/4	0.179	0.177
***Sensory Neuropathy***		
None vs. Grade 1/2 vs. Grade 3/4	0.218	0.115
***Skin Toxicity***		
None vs. Grade 1/2 vs. Grade 3/4	0.109	0.396
***Alopecia***		
None vs. Grade 1 vs. Grade 2	0.107	0.404
***Pain***		
None vs. Mild vs. Moderate vs. Severe	0.176	0.176

Dependent factor: survival threshold; independent factors: disease group, age, sex, psychological morbidity, toxicity, and pain.

## Discussion

Major goals of palliative chemotherapy are to improve patients’ subjective well-being and to prolong survival. In contrast to the latter, the patients’ perspectives on benefits, toxicities and burden are less frequently studied although important for decision making in several aspects – the shared decision making of the patient with his or her treating physician and the allocation of financial or other resources.

In our study population, acne, fatigue, and gastrointestinal (GI) side effects were among the most frequently PRO- grade 3/4 toxicity, with fatigue being the most severe burden as reported by 14.3% of all patients. It is known that skin and GI toxicity are typical grade 3/4 adverse events in multiple phase III chemotherapy trials [5,25-27]. On the other hand, fatigue has rarely been reported to be a major toxicity in such trials. This may be because fatigue might be a symptom of the underlying malignant disease and therefore cannot be attributed to therapy alone [28,29]. It is known that 80.0% to 99.0% of patients undergoing chemotherapy report fatigue at some point [30]. Our study showed that fatigue was worse than expected in one third of patients. These findings are in agreement with data from Olver et al [31], who compared patients’ pre-treatment expectations of toxicities with post-chemotherapy experiences. They found that “feeling tired” was among the side effects that occurred in more patients than was initially expected. Interesting in this regard is the fact that fatigue is not detailed on most commonly used consent forms [32]. We believe that fatigue deserves more attention when toxicity of palliative treatment and symptoms of disease are explained to the patient.

Overall, self-reported grade 3/4 toxicity and severe burden rates remained relatively low. Patients seemed to have been well informed about most adverse events (apart from fatigue) prior to treatment. Expectations of side effects were higher than their actual occurrence which supports the findings in other studies [31].

The median actual overall survival in our study was 30.0 months in the CRC and 19.0 months in the non-CRC group. This result exceeds the median overall survival observed in most phase III trials including metastatic CRC and non-CRC cancer [2-5]. A length of time bias together with the fact that we only included patients who had already completed a minimum of three months of palliative chemotherapy, possibly consistent with a potential therapy benefit, may contribute to this finding.

In the current study, patients’ anticipated median survival thresholds were longer (CRC 36.0 months; non-CRC 18.0 months) than described in previous reports [7-11]. This might be influenced by the different surveying methods. Most prior studies used hypothetical situations and classical time trade-off techniques where patients were encouraged to choose between a given number of months, time spans, or risk reduction they would consider a threshold for therapy. In scenarios with mild toxicity, participants would choose therapy for median benefits ranging from 1.5 months [10] to 4.5 months [11]. For more intensive treatment situations, expected survival benefits ranged from 7.5 months [10] up to 12 months [8]. The authors of those studies concluded that patients were willing to undergo treatment for small survival benefits. In contrast, patients in our study were asked to state the exact survival thresholds for which they would repeat chemotherapy. Therefore discrepancies might reflect the difference between preferences in a hypothetical scenario and those in a personally relevant clinical situation [14]. The anticipated survival threshold in our study varied widely (zero to 340 months) which confirms the fact that patients’ expectations are extremely heterogeneous [8,11,33].

To our knowledge, this is the first study comparing patients’ survival expectations with their actual survival. Interestingly, the patients’ expected survival significantly exceeded their actual survival time (*p*=0.003). In a study by Brundage et al, the expectations of more than half of the participants exceeded the estimated, realistic survival benefit defined prior to survey [12]. Chu et al found that 60.0% of their advanced NSCLC patients would prefer a maximum extension of survival with the acceptance of high toxicity [13]. Additionally, in a Korean survey, patients with metastatic solid cancers requested a two-fold longer survival threshold than previous studies had suggested [14]. Similar to our findings of higher expectations in CRC patients, the recently published study of Weeks and colleagues demonstrated that patients’ expectations from palliative chemotherapy are frequently unrealistic and often even include cure [34].

Our results might have been biased by the construction of the questionnaire used in the study. Patients were asked about toxicity and burden from therapy first and then to state the anticipated survival threshold. They might have demanded higher thresholds from therapy after being forced to recall toxicity. Conceivably, some patients might have rather projected their wish for overall survival than survival threshold. Furthermore, the patient group was self-selecting as only 72.3% of the participants would answer the survival threshold question at all. As our patient population had completed a median time of six months of therapy prior to study, which might be interpreted as evidence of a therapy benefit, patients with rapid progression or high levels of toxicity forcing an early discontinuance of therapy might have had an entirely different view on survival threshold. Other patients might have been overwhelmed by the question as it is known that many palliative patients do not give any consideration to end-of-life issues [35,36].

Factors independently influencing the anticipated survival threshold were disease group, depression, and diarrhea. As depression is known to be positively correlated with hopelessness [37,38] this might be the explanation why patients in our study with higher HADS-D scores (possible depression) were more willing to repeat therapy for lower thresholds than patients with lower scores. Patients in the non-CRC group were more likely to show abnormal scores in any subscale of HADS, compatible with psychological morbidity. This is in agreement with a number of other studies showing that cancer types with poor prognosis such as lung, pancreatic, or head and neck cancers are associated with the highest rates of psychological distress [39,40]. As in several other trials, gender [9-12] as well as previous therapy experience [12] did not influence the anticipated survival thresholds. The influence of age on anticipated survival is controversial in the literature. Some authors claim that older patients expect greater benefits [11,12]. Other studies as well as our own did not show any association between patient age and anticipated survival benefits from palliative chemotherapy [10,13].

## Conclusions

In conclusion, our data show that patients seem to be sufficiently informed about possible adverse events during chemotherapy as occurrence and extent were similar to reported phase III trials. However, fatigue deserves more attention when treatment and symptoms of disease are discussed with the patient. Patients’ expectations from survival with palliative chemotherapy are higher than previously described and exceed their actual survival. Even though new therapies have achieved significant improvements in overall survival, such expectations cannot be met yet. More detailed discussions about survival benefits are necessary prior to therapy [41] to facilitate shared decision making.

## Competing interests

G. Folprecht (not directly related to the current study):

Advisory boards: Merck KGaA, Roche, Bristol-Myes-Squibb

Study grants: Merck KGaA

Lecture honoraria: Merck KGaA, Roche, Novartis, Sanofi-Aventis, Amgen

All remaining authors have declared no conflict of interests.

## Authors’ contributions

MM: study design, data acquisition, data analysis and interpretation, manuscript writing, KT: data analysis and interpretation, manuscript writing, G. Ehninger: administrative support, study design, quality control of data and algorithms AR: statistical analysis, quality control of data and algorithms, BH: study design, data analysis and interpretation, U. S: study design, data interpretation, quality control of data and algorithms, GF: study concepts and design, data acquisition, analysis and interpretation, quality control of data and algorithms, statistical analysis. All authors read and approved the final manuscript.

## Authors’ information

Interim results of the current study were presented at 2011 ASCO Gastrointestinal Cancers Symposium, January 20^th^ - 23^rd^ 2011, San Francisco, General Poster Session

## Pre-publication history

The pre-publication history for this paper can be accessed here:

http://www.biomedcentral.com/1471-2407/13/66/prepub

## Supplementary Material

Additional file 1: Table S1B Chemotherapy at time of study and prior to study. **Figure S2B. **PROs in toxicity by disease group. Green: All Patients, Red: CRC, Blue: Non-CRC. **Figure S4. **Questionnaire in German with English translation. Click here for file

## References

[B1] Ferlay J Shin HR Bray F Forman D Mathers C Parkin DM GLOBOCAN 2008 v2.0, Cancer Incidence and Mortality Worldwide: IARC CancerBase No. 10 [Internet] 2010 Lyon, France: International Agency for Research on Cancer Available from: http://globocan.iarc.fr/, Assessed on January 26, 2011

[B2] Kopetz S Chang GJ Overman MJ Eng C Sargent DJ Larson DW Improved survival in metastatic colorectal cancer is associated with adoption of hepatic resection and improved chemotherapy J Clin Oncol 2009 27 3677 3683 10.1200/JCO.2008.20.5278 19470929 PMC2720081

[B3] Van Cutsem E Moiseyenko VM Tjulandin S Majlis A Costenla M Boni C Phase III study of docetaxel and cisplatin plus fluorouracil compared with cisplatin and fluorouracil as first-line therapy for advanced gastric cancer: a report of the V325 study group J Clin Oncol 2006 24 4991 4997 10.1200/JCO.2006.06.8429 17075117

[B4] Sandler A Gray R Perry MC Brahmer J Schiller JH Dowlati A Paclitaxel–carboplatin alone or with bevacizumab for non–small-cell lung cancer N Engl J Med 2006 355 2542 2550 10.1056/NEJMoa061884 17167137

[B5] Vermorken JB Mesia R Rivera F Remenar E Kawecki A Rottey S Platinum-based chemotherapy plus cetuximab in head and neck cancer N Engl J Med 2008 359 1116 1127 10.1056/NEJMoa0802656 18784101

[B6] United States Food and Drug Administration Guidance for industry (draft). Patient-reported outcome measures: use in medical product development to support labeling claims 2006 Rockville (MD): U.S: Department of Health and Human Services

[B7] Matsuyama R Reddy S Smith TJ Why do patients choose chemotherapy near the end of life? A review of the perspective of those facing death from cancer J Clin Oncol 2006 24 3490 3496 10.1200/JCO.2005.03.6236 16849766

[B8] Slevin ML Stubbs L Plant HJ Wilson P Gregory WM Armes PJ Attitudes to chemotherapy: comparing views of patients with cancer with those of doctors, nurses, and general public BMJ 1990 300 1458 1460 10.1136/bmj.300.6737.1458 2379006 PMC1663147

[B9] Love N Bylund C Meropol NJ Marshall JL Curley SA Ellis LM How Well Do We Communicate with Patients Concerning Adjuvant Systemic Therapy? A Survey of 150 Colorectal Cancer Survivors 2007 Proceedings of the ASCO Gastrointestinal Cancers Symposium, Abstract No: 239

[B10] Balmer CE Thomas P Osborne RJ Who wants second–line, palliative Chemotherapy? Psychooncology 2001 10 410 418 10.1002/pon.538 11536419

[B11] Silvestri G Pritchard R Welch HG Preferences for chemotherapy in patients with advanced non-small cell lung cancer: descriptive study based on scripted interviews BMJ 1998 317 771 775 10.1136/bmj.317.7161.771 9740561 PMC28665

[B12] Brundage MD Feldman-Stewart D Cosby R Gregg R Dixon P Youssef Y Cancer patients’ attitudes toward treatment options for advanced non-small cell lung cancer: implications for patient education and decision support Patient Educ Couns 2001 45 149 157 10.1016/S0738-3991(01)00155-0 11687329

[B13] Chu DT Kim SW Hsu HK Cok G Roubec J Patil S Patients attitudes towards chemotherapy and survival: a prospective observational study in advanced non-small cell lung cancer Lung Cancer 2009 66 250 256 10.1016/j.lungcan.2009.01.022 19264374

[B14] Kim MK Lee JL Hyun MS Do YR Song HS Kim JG Palliative chemotherapy preferences and factors that influence patient choice in incurable advanced cancer Jpn J Clin Oncol 2008 38 64 70 10.1093/jjco/hym147 18238880

[B15] Walling A Lorenz KA Dy SM Naeim A Sanati H Asch SM Evidence-based recommendations for information and care planning in cancer care J Clin Oncol 2008 26 3896 3902 10.1200/JCO.2007.15.9509 18688058

[B16] Cancer Therapy Evaluation Program, Common Terminology Criteria for Adverse Events, Version 3.0, DCTD, NCI, NIH, DHHS March 31, 2003 (http://ctep.cancer.gov), Publish Date: June 10, 2003

[B17] Serlin RC Mendoza TR Nakamura Y Edwards KR Cleeland CS When is cancer pain mild, moderate or severe? Grading pain severity by its interference with function Pain 1995 61 277 284 10.1016/0304-3959(94)00178-H 7659438

[B18] Sigmond AS Snaith RP The hospital anxiety and depression scale Acta Psychiatr Scand 1983 67 361 370 10.1111/j.1600-0447.1983.tb09716.x 6880820

[B19] Bjelland I Dahl AA Haug TT Neckelmann D The validity of the hospital anxiety and depression scale an updated literature review J Psychosom Res 2002 52 69 77 10.1016/S0022-3999(01)00296-3 11832252

[B20] Likert R A technique for the measurement of attitudes Arch Psych 1932 140 1 55

[B21] Group CCC Simmonds PC Palliative chemotherapy for advanced colorectal cancer: systematic review and meta – analysis BMJ 2000 321 531 535 10968812 10.1136/bmj.321.7260.531 PMC27466

[B22] Wagner AD Grothe W Haerting J Kleber G Grothey A Fleig WE Chemotherapy in advanced gastric cancer: a systematic review and meta - analysis based on aggregate data J Clin Oncol 2006 24 2903 2909 10.1200/JCO.2005.05.0245 16782930

[B23] Rapp E Pater JL Willan A Cormier Y Murray N Evans WK Chemotherapy Can prolong survival in patients with advanced non-small-cell lung cancer-report of a Canadian multicenter randomized trial J Clin Oncol 1988 6 633 641 2833577 10.1200/JCO.1988.6.4.633

[B24] Stell PM Morton RP Singh SD Squamous carcinoma of the head and neck: the untreated patient Clin Otolaryngol Allied Sci 1983 8 7 13 10.1111/j.1365-2273.1983.tb01665.x 6831756

[B25] Van Cutsem E Köhne CH Hitre E Zaluski J Chang Chien CR Makhson A Cetuximab and chemotherapy as initial treatment for metastatic colorectal cancer N Engl J Med 2009 360 1408 1417 10.1056/NEJMoa0805019 19339720

[B26] Pirker R Pereira JR Szczesna A von Pawel J Krzakowski M Ramlau R Cetuximab plus chemotherapy in patients with advanced non-small-cell lung cancer (FLEX): an open-label randomised phase III trial Lancet 2009 373 1525 1531 10.1016/S0140-6736(09)60569-9 19410716

[B27] Cunningham D Starling N Rao S Iveson T Nicolson M Coxon F Capecitabine and oxaliplatin for advanced esophagogastric cancer N Engl J Med 2008 358 36 46 10.1056/NEJMoa073149 18172173

[B28] Narayanan V Koshy C Fatigue in cancer: a review of literature Indian J Palliat Care 2009 15 19 25 10.4103/0973-1075.53507 20606851 PMC2886215

[B29] Ahlberg K Ekman T Gaston-Johansson F Mock V Assessment and management of cancer-related fatigue in adults Lancet 2003 362 640 650 10.1016/S0140-6736(03)14186-4 12944066

[B30] Curt GA Breitbart W Cella D Groopman JE Horning SJ Itri LM Impact of cancer-related fatigue on the lives of patients: new findings from the fatigue coalition Oncologist 2000 5 353 360 10.1634/theoncologist.5-5-353 11040270

[B31] Olver IN Taylor AE Whitford HS Relationships between patients’ pre-treatment expectations of toxicities and post chemotherapy experiences Psychooncology 2005 14 25 33 10.1002/pon.804 15386792

[B32] Sauer H Basisinformation zum Aufklärungsgespräch Zytostatische Chemotherapie 2009 Erlangen: proCompliance in Thieme GmbH

[B33] Brundage MD Davidson JR Mackillop WJ Trading treatment toxicity for survival in locally advanced non-small cell lung cancer J Clin Oncol 1997 15 330 340 8996160 10.1200/JCO.1997.15.1.330

[B34] Weeks JC Catalano PJ Cronin A Finkelman MD Mack JW Keating NL Patient’s expectations about effects of chemotherapy for advanced cancer N Engl J Med 2012 367 1616 1625 10.1056/NEJMoa1204410 23094723 PMC3613151

[B35] Chow E Andersson L Wong R Vachon M Hruby G Franssen E Patients with advanced cancer: a survey of the understanding of their illness and expectations from palliative radiotherapy for symptomatic metastases Clin Oncol 2001 13 204 208 10.1053/clon.2001.9255 11527297

[B36] Wright AA Zhang B Ray A Mack JW Trice E Balboni T Associations between end-of-life discussions, patient mental health, medical care near death, and caregiver bereavement adjustment JAMA 2008 300 1665 1673 10.1001/jama.300.14.1665 18840840 PMC2853806

[B37] Mystakidou K Tsilika E Parpa E Athanasouli P Galanos A Anna P Illness-Related Hopelessness in Advanced Cancer Influence of anxiety, depression, and preparatory grief Arch Psychiatr Nurs 2009 23 138 147 10.1016/j.apnu.2008.04.008 19327556

[B38] Pessin H Rosenfeld B Breitbart W Assessing psychological distress near the end of life Am Behav Sci 2002 46 357 372 10.1177/000276402237769

[B39] Weis J Boehncke A Psychische Komorbidität bei Krebserkrankungen Bundesgesundheitsbl 2011 54 46 51 10.1007/s00103-010-1184-y 21246328

[B40] Zabora J Brintzenhofeszoc K Jacobsen P Curbow B Piantadosi S Hooker C A new psychosocial screening instrument for use with cancer patients Psychosomatics 2001 42 241 246 10.1176/appi.psy.42.3.241 11351113

[B41] Smith TJ Hillner BE Bending the cost curve in cancer care NEJM 2011 364 2060 2065 10.1056/NEJMsb1013826 21612477 PMC4042405

