# Supplementary material for: Patients’ perspectives on palliative chemotherapy of colorectal and non - colorectal cancer: a prospective study in a chemotherapy- experienced population
Source: BMC Cancer. 2013 Feb 7;13:66. doi: 10.1186/1471-2407-13-66 (PMC3632496; doi:10.1186/1471-2407-13-66)
Supplement: Additional file 1: Table S1B — Chemotherapy at time of study and prior to study. Figure S2B. PROs in toxicity by disease group. Green: All Patients, Red: CRC, Blue: Non-CRC. Figure S4. Questionnaire in German with English translation. [file 1471-2407-13-66-S1.doc]

**Table S1B. (proposed for web appendix) Chemotherapy at time of study and prior to study**

| **Chemotherapy** | **CRC** | | **Non-CRC** | |
| --- | --- | --- | --- | --- |
|  | CTx at time of study | CTx prior to study | CTx at time of study | CTx prior to study |
| n | 58 | 58 | 76 | 76 |
|  |  |  |  |  |
| Platinum-containing ± antibody | 18 | 17 | 41 | 29 |
|  |  |  |  |  |
| Oxaliplatin | 18 | 17 | 16 | 12 |
| Cis-/Carboplatin | 0 | 0 | 25 | 17 |
|  |  |  |  |  |
| Irinotecan-containing ± antibody | 37 | 13 | 3 | 5 |
|  |  |  |  |  |
| Fluoropyrimidine only ± antibody/TKI | 7 | 6 | 3 | 2 |
|  |  |  |  |  |
| Gemcitabine-containing ± TKI | 0 | 0 | 21 | 13 |
|  |  |  |  |  |
| Taxane-containing | 0 | 0 | 11 | 9 |
|  |  |  |  |  |
| Other * | 0 | 0 | 2 | 1 |
|  |  |  |  |  |
| Anti-EGFR | 16 | 8 | 9 | 5 |
| Anti-VEGF | 24 | 10 | 1 | 1 |
| Tyrosine kinase inhibitors | 0 | 0 | 10 | 2 |
|  |  |  |  |  |
| None | 0 | 28 | 0 | 36 |
|  |  |  |  |  |

All components of any CTx-regimen were counted. For CTx-regimen with more than one drug, all drugs were counted separately. Finally, two groups were formed: platinum- vs. non-platinum-containing therapy.

* Other cytotoxic drugs were pemetrexed and topotecan

**Figure S2B.** **(proposed for web appendix) PROs in toxicity by disease group**

Green: All Patients, Red: CRC, Blue: Non-CRC

* Patients’ expectations in comparison to informed consent: much less, less, more,

and much more than expected.

° Grades of patient-reported toxicity for alopecia are: none, grade 1, grade 2

**Figure S4. (proposed for web appendix) Questionnaire in German with English translation**

Liebe Patientinnen und Patienten,

In der Medizinischen Klinik I des Universitätsklinikums Dresden untersuchen wir wie sich Patienten mit einer Chemotherapie fühlen und welche Nebenwirkungen sie am meisten spüren. Für die Untersuchung sind wir auf Ihre Erfahrungen und Hilfe angewiesen.

Ziel dieser Studie ist es, das Arzt-Patienten-Gespräch zu verbessern, den Einsatz von chemotherapeutischen Medikamenten zu optimieren und die Behandlung von Nebenwirkungen genauer zu steuern.

Daher wären wir Ihnen dankbar, wenn Sie den Fragebogen ausfüllen und uns damit unterstützen.

Im Folgenden werden Ihnen Fragen gestellt, die Sie meist einfach durch ein entsprechendes Ankreuzen in den vorgegebenen Feldern beantworten können. Überlegen Sie bitte nicht lange, sondern wählen Sie die Antwort aus, die Ihnen auf Anhieb am besten erscheint. Richtige oder falsche Antworten gibt es nicht.

Für die Auswertung ist es wichtig, dass Sie möglichst alle Fragen ausfüllen.

Nachdem Sie den Fragebogen ausgefüllt und in den Umschlag getan haben, verschließen Sie diesen bitte. Geben Sie den Umschlag in der Tagesklinik oder auf der Station ab.

Ihre Angaben unterliegen dem Datenschutz, d.h. sie werden vertraulich und anonym behandelt. Ärzte und Pflegepersonal werden keinen Einblick in Ihre Angaben erhalten.

Page 1

**Einwilligungserklärung zum Datenschutz**

**Ich erkläre mich damit einverstanden, dass im Rahmen dieser Befragung**

**erhobene Daten auf Fragebögen und elektronischen Datenträgern aufgezeichnet und in verschlüsselter Form (ohne Namens- und Initialiennennung) wissenschaftlich ausgewertet werden dürfen.**

**Außerdem erkläre ich mich damit einverstanden, dass autorisierte**

**und zur Verschwiegenheit verpflichteter Beauftragte der Ethik-Kommission in meine beim Prüfarzt vorhandenen personenbezogenen Daten Einsicht nehmen dürfen, soweit dies für die Überprüfung der Ergebnisse notwendig sein sollte.**

**Für diese Maßnahme entbinde ich den Arzt von der ärztlichen Schweigepflicht.**

**Name, Vorname:**

**Geburtsdatum :**

**Dresden, den Unterschrift**

Page 2

Im Folgenden möchten wir gern wissen, welche Veränderungen Ihnen während der

Chemotherapie an Ihrem Körper aufgefallen sind.

Dann sollen Sie sich bitte auch an das Aufklärungsgespräch mit Ihrem Arzt vor Therapiebeginn erinnern. Waren die Nebenwirkungen anders als von Ihnen nach dem Gespräch erwartet?

Des Weiteren bitten wir Sie festzulegen, wie diese Veränderungen Sie in Ihrem täglichen Leben belastet haben.

*Bitte kreuzen Sie dazu das für Sie zutreffende Kästchen an und zeichnen Sie auf dem vorgegebenen Strahl die Intensität ein.*

*Zuerst ein Beispiel:*

*Wie stark haben diese Haarveränderungen Sie belastet?*

*„Da ich kaum Haarausfall hatte, fühlte ich mich nur wenig beeinträchtigt, deshalb muss ich die Stärke der Beeinträchtigung auf dem Strahl wie folgt eintragen:“*

|  | **X** |  |  |  |  |  |  |  |  |
| --- | --- | --- | --- | --- | --- | --- | --- | --- | --- |

0 1 2 3 4 5 6 7 8 9 10

Gar nicht (0) Stärkste vorstellbare

Belastung (10)

Page 3

| **1. SCHMERZ**  Hatten Sie Schmerzen während der Chemotherapie?  Bitte geben Sie auf einer Skala von 0 bis 10 die Stärke der Schmerzen an.   |  |  |  |  |  |  |  |  |  |  | | --- | --- | --- | --- | --- | --- | --- | --- | --- | --- |   0 1 2 3 4 5 6 7 8 9 10 |  |
| --- | --- | --- | --- | --- | --- | --- | --- | --- | --- | --- | --- |
| **2. HAARVERÄNDERUNGEN**  a) Wie haben sich Ihre Haare verändert?   | □ | Gar nicht. | | --- | --- | | □ | Sie sind ausgedünnt. | | □ | Sie sind komplett ausgefallen. | |  |
| **Bitte beantworten Sie die folgenden Fragen auch wenn Sie keine Haarveränderungen hatten:** | |
| b) Waren die Haarveränderungen anders als von Ihnen nach dem Aufklärungsgespräch mit Ihrem Arzt erwartet?   | □ | Es gab viel weniger Haarveränderungen als erwartet. | | --- | --- | | □ | Etwas weniger Haarveränderungen als erwartet. | | □ | Genauso wie erwar | | □et. | Etwas mehr Haarveränderungen als erwartet. | | □ | Viel mehr Haarveränderungen als erwarte | | . |  | | c) Wie stark fühlten Sie sich durch diese Haarveränderungen belastet?   |  |  |  |  |  |  |  |  |  |  | | --- | --- | --- | --- | --- | --- | --- | --- | --- | --- |   0 1 2 3 4 5 6 7 8 9 10 |
| **3. MÜDIGKEIT/ABGESCHLAGENHEIT**  a) Fühlten Sie sich abgeschlagen und müde?   | □ | Nein | | --- | --- | | □ | Etwas mehr als normal. | | □ | Mittelmäßig. Ich hatte Probleme, meine täglichen Aufgaben zu schaffen. | | □ | Schwer. Ich konnte meine täglichen Aufgaben nicht | | □mehr bewältigen. | Stark behindernd. | |  |
| **Bitte beantworten Sie folgende Fragen auch wenn Sie sich nicht müde und abgeschlagen gefühlt haben:** | |
| b) Waren Müdigkeit und Abgeschlagenheit anders als von Ihnen nach dem Aufklärungsgespräch mit Ihrem Arzt erwartet?   | □ | Viel weniger Müdigkeit und Abgeschlagenheit als erwartet. | | --- | --- | | □ | Etwas weniger Müdigke | | □t und Abgeschlagenheit als erwartet. | Genauso wie erwartet. | | □ | Etwas mehr Müdigkeit und Abgeschlagenheit als erwartet. | | □ | Viel mehr Müdigkeit und Abges |   hlagenheit als erwartet. | c) Wie stark fühlten Sie sich durch diese Müdigkeit und Abgeschlagenheit belastet?   |  |  |  |  |  |  |  |  |  |  | | --- | --- | --- | --- | --- | --- | --- | --- | --- | --- |   0 1 2 3 4 5 6 7 8 9 10  Page 4 |

| **4. ÜBELKEIT**  a) Wie oft war Ihnen übel?   | □ | Nie. | | --- | --- | | □ | Ich hatte Appetitsverl | | □st, musste meine Essgewohnheiten aber nicht ändern. | Ich konnte nur wenig essen, habe aber kaum an Gewicht verloren. | | □ | Ich habe kaum Nahrung oder Flüssigkeiten zu mir nehmen können. | | b) Haben Sie aufgrund der Übelkeit Flüssigkeit über die Vene bekommen?   | □ | Nein. | | --- | --- | | □ | Ja, für einen Tag. | | □ | Ja, für mehrere Tage. | |
| --- | --- | --- | --- | --- | --- | --- | --- | --- | --- | --- | --- | --- | --- | --- | --- |
| c) Ist Ihnen aufgrund der Übelkeit eine Magensonde gelegt worden?   | □ | Nein. | | --- | --- | | □ | Ja. | |  |
| **Bitte beantworten Sie folgende Fragen auch wenn Ihnen nicht übel war:** | |
| d) War die Übelkeit anders als von Ihnen nach dem Aufklärungsgespräch mit Ihrem Arzt erwartet?   | □ | Viel weniger Übelkeit als erwartet. | | --- | --- | | □ | Etwas weniger Übelkeit als erwartet. | | □ | Genauso wie erwartet. | | □ | Etwas mehr Übelkeit als e | | □wartet. | Viel mehr Übelkeit als erwartet. | | e) Wie stark fühlten Sie sich durch die Übelkeit belastet?   |  |  |  |  |  |  |  |  |  |  | | --- | --- | --- | --- | --- | --- | --- | --- | --- | --- |   0 1 2 3 4 5 6 7 8 9 10 |
| **5. ERBRECHEN**  a) Wie oft mussten Sie sich erbrechen?   | □ | Nie. | | --- | --- | | □ | Höchstens 1 Mal pr | | □ Tag. | 2 bis 5 Mal pro Tag. | | □ | Mehr als 6 Mal pro Tag. | |  |  | | b) Haben Sie aufgrund des Erbrechens Flüssigkeit über die Vene bekommen?   | □ | Nein. | | --- | --- | | □ | Ja, für einen Tag. | | □ | Ja, für mehrere Tage. | |  |  | |
| **Bitte beantworten Sie folgende Fragen auch wenn Sie sich nicht erbrechen mussten:** | |
| c) War das Erbrechen anders als von Ihnen nach dem Aufklärungsgespräch mit Ihrem Arzt erwartet?   | □ | Viel weniger Erbrechen als erwartet. | | --- | --- | | □ | Etwas weniger Erbrechen als erwartet | | □ | Genauso wie erwartet. | | □ | Etw | | □s mehr Erbrechen als erwartet. | Viel mehr Erbrechen als erwartet. | |  |  | | d) Wie stark fühlten Sie sich durch das Erbrechen belastet?   |  |  |  |  |  |  |  |  |  |  | | --- | --- | --- | --- | --- | --- | --- | --- | --- | --- |   0 1 2 3 4 5 6 7 8 9 10  Page 5 |

| **6. DURCHFALL**  a) Wie oft hatten Sie Durchfall?   | □ | Nie. | | --- | --- | | □ | Höchstens 4 Mal mehr pro Tag als ohne Chemotherapie. | | □ | Zwischen 4 bis 6 Mal mehr pro Tag als ohne Chemotherapie. | | □ | Häufiger als 7 Mal mehr pro Tag. | | b) Für Patienten mit künstlichem Darmausgang (Stoma):  Wie hat sich Ihr Stuhl verändert?   | □ | Gar nicht. | | --- | --- | | □ | Es sammelte sich etwas mehr Stuhl im künstlichen Darmausgang. | | □ | Es sammelte sich mehr Stuhl im künstlichen Darmausgang. | | □ | Es sammelte sich sehr viel mehr Stuhl im künstlichen |   Darmausgang. | |  |
| --- | --- | --- | --- | --- | --- | --- | --- | --- | --- | --- | --- | --- | --- | --- | --- | --- | --- | --- | --- |
| c) Haben Sie aufgrund des Durchfalls Flüssigkeit über die Vene bekommen?   | □ | Nein. | | --- | --- | | □ | Ja, für einen Tag. | | □ | Ja, für mehrere Tage. | | d) Hatten Sie aufgrund des Durchfalls Probleme, Ihre täglichen Aufgaben zu bewältigen?   | □ | Nei | | --- | --- | | □. | Ja. | | □ | Ich konnte meine täglichen Aufgaben gar nicht bewältigen. | | |  |
| e) Mussten Sie aufgrund des Durchfalls ins Krankenhaus?   | □ | Nein. | | --- | --- | | □ | Ja. | |  | |  |
| **Bitte beantworten Sie folgende Fragen auch wenn Sie keinen Durchfall hatten:** | | | |
| f) War der Durchfall anders als von Ihnen nach dem Aufklärungsgespräch mit Ihrem Arzt erwartet?   | □ | Viel weniger Durchfall als erwartet. | | --- | --- | | □ | Etwas weniger Durchfall als erwartet. | | □ | Genauso wie erwartet. | | □ | Etwas mehr Durchfall als er | | □artet. | Viel mehr Durchfall als erwartet. | | | g) Wie stark fühlten Sie sich durch den Durchfall belastet?   |  |  |  |  |  |  |  |  |  |  | | --- | --- | --- | --- | --- | --- | --- | --- | --- | --- |   0 1 2 3 4 5 6 7 8 9 10 | |
|  | | |  |
| Page 6 | | |  |
|  |  | |  |
| **7. ENTZÜNDUNGEN IM MUND/RACHEN**  a) Hatten Sie eine Entzündung im Mund-Rachen-Bereich?   | □ | Nein. | | --- | --- | | □ | Ja. | | b) Hatten Sie aufgrund der Entzündung Probleme beim Essen?   | □ | Nein. | | --- | --- | | □ | Ich hatte geringe Schwierigkeiten beim Essen. | | □ | Ich konnte kaum noch etwas essen. | | |  |
| c) Hatten Sie aufgrund der Entzündung Probleme beim Atmen?   | □ | Nein | | --- | --- | | □ | Ich hatte geringe Schwierigkeiten beim Atmen. | | □ | Ich hatte größere Schwierigkeiten beim Atmen. | |  | |  |
| **Bitte beantworten Sie folgende Fragen auch wenn Sie keine Entzündung hatten:** | | |  |
| d) Hatten Sie aufgrund der Entzündung Probleme, Ihre täglichen Aufgaben zu bewältigen   | □ | Nein. | | --- | --- | | □ | Ja. | | □ | Ich konnte meine täglichen Aufgaben gar nicht bewältigen. | | e) Waren die Entzündungen im Mund-Rachen-Raum anders als von Ihnen nach dem Aufklärungsgespräch mit Ihrem Arzt erwartet?   | □ | Viel weniger Entzündungen a | | --- | --- | | □s erwartet. | Etwas weniger Entzündungen als erwartet. | | □ | Genauso wie erwartet. | | □ | Etwas mehr Entzündungen als erwartet. | | □ | Viel mehr Entzündungen als erwartet. | | |  |
| f) Wie stark fühlten Sie sich durch die Entzündungen im Mund-Rachen-Raum belastet?   |  |  |  |  |  |  |  |  |  |  | | --- | --- | --- | --- | --- | --- | --- | --- | --- | --- |   0 1 2 3 4 5 6 7 8 9 10 |  | |  |
| **8. EMPFINDUNGSSTÖRUNGEN**  a) Hatten Sie Empfindungsstörungen (Taubheitsgefühl) an den Händen und/oder Füßen?   | □ | Nein. | | --- | --- | | □ | Ja. | | □ | Ja und ich fühlte mich s |   ark behindert. | b) Hatten Sie aufgrund der Empfindungsstörungen Probleme, Ihre täglichen Aufgaben zu bewältigen?   | □ | Nein. | | --- | --- | | □ | Ja. | | □ | Ich konnte meine täglichen Aufgaben gar nicht bewältigen. | | |  |
| **Bitte beantworten Sie folgende Fragen auch wenn Sie keine Empfindungsstörungen hatten:**  Page 7 | | |  |
| c) Waren die Empfindungsstörungen an den Händen und/oder Füßen anders als von Ihnen nach dem Aufklärungsgespräch mit Ihrem Arzt erwartet?   | □ | Viel weniger | | --- | --- | | □Empfindungsstörungen als erwartet. | Etwas we | | □iger Empfindungsstörungen als erwartet. | Genauso wie erwartet. | | □ | Etwas mehr Empfindungsstörungen als erwartet. | | □ | Viel mehr Empfindungsstörungen als erwartet. | | d) Wie stark fühlten Sie sich durch die Empfindungsstörungen an den Händen und/oder Füßen belastet?   |  |  |  |  |  |  |  |  |  |  | | --- | --- | --- | --- | --- | --- | --- | --- | --- | --- |   0 1 2 3 4 5 6 7 8 9 10 | |  |
| **9. HAUT**  a) Haben Sie während der Therapie eine vermehrte Pickelbildung (Akne) bei sich festgestellt?   | □ | Nein. | | --- | --- | | □ | Ja. | | b) Mussten Sie deshalb behandelt werden?   | □ | Nein. | | --- | --- | | □ | Ja. | | |  |
| c) Trat diese Pickelbildung zusammen mit Schmerzen oder Entstellung auf?   | □ | Nein. | | --- | --- | | □ | Ja. | |  | |  |
| **Bitte beantworten Sie die folgenden Fragen auch wenn bei Ihnen keine vermehrte Pickelbildung aufgetreten ist:** | | |  |
| d) War die Pickelbildung an der Haut anders als von Ihnen nach dem Aufklärungsgespräch mit Ihrem Arzt erwartet?   | □ | Viel weniger Pickelbildung als erwartet. | | --- | --- | | □ | Etwas weniger Pickelbildung als erwartet. | | □ | G | | □nauso wie erwartet. | Etwas mehr Pick | | □lbildung als erwartet. | Viel mehr Pickelbildung als erwartet. | | e) Wie stark fühlten Sie sich durch die Pickelbildung an der Haut belastet?   |  |  |  |  |  |  |  |  |  |  | | --- | --- | --- | --- | --- | --- | --- | --- | --- | --- |   0 1 2 3 4 5 6 7 8 9 10 | |  |
| **10. ANDERE NEBENWIRKUNGEN**  a) Falls bei Ihnen andere Nebenwirkungen auftraten, die oben nicht genannt sind, tragen Sie diese bitte hier ein. | | |  |
| b) Hatten Sie aufgrund dieser Nebenwirkungen Probleme, Ihre täglichen Aufgaben zu bewältigen?   | □ | Nein. | | --- | --- | | □ | Ja. | | □ | Ich konnte meine täglichen Aufgaben  gar nicht bewältigen. | | c) Waren diese Nebenwirkungen lebensbedrohlich?   | □ | Nein. | | --- | --- | | □ | Ja. |   Page 8 | |  |

| 11. Nachdem Sie in den letzten Wochen und Monaten aufgrund Ihrer Krebstherapie eine schwierige und anstrengende Zeit erlebt haben, möchten wir gern wissen, wie Sie zum heutigen Tag darüber denken?  1.  Wenn Sie heute vor der Entscheidung stehen würden sich nochmals dieser Chemotherapie mit ihren Wirkungen, aber auch Nebenwirkungen zu unterziehen, würden Sie es tun?   | □ | Nein | | --- | --- | | □ | Eher nicht | | □ | Weiß ich nicht | | □ | Eher ja | | □ | Ja | |
| --- | --- | --- | --- | --- | --- | --- | --- | --- | --- | --- |
| Wenn Sie an die Belastung der Chemotherapie denken: Um welche Zeit müssten Sie mindestens länger leben können, damit Sie sich dieser Therapie nochmals unterziehen würden?  2.  Der Gewinn an Lebenszeit müsste mindestens Monat(e) betragen,  sonst würden sich die Belastungen nicht lohnen. |
| Bitte beantworten Sie die Fragen auf der nächsten Seite.  Page 9 |

| **HADS** |
| --- |

| 12. Wir bitten Sie jedoch, jede Frage zu beantworten, und zwar so, wie es für Sie persönlich **in der letzten Woche** am ehesten zutraf. |
| --- |

| a) Ich fühle mich angespannt oder überreizt  □ meistens  □ oft  □ von Zeit zu Zeit/gelegentlich  □ überhaupt nicht | h) Ich fühle mich in meinen Aktivitäten gebremst  □ fast immer  □ sehr oft  □ manchmal  □ überhaupt nicht |
| --- | --- |
| b) Ich kann mich heute noch so freuen wie früher  □ ganz genau so  □ nicht ganz so sehr  □ nur noch ein wenig  □ kaum oder gar nicht | i) Ich habe manchmal ein ängstliches Gefühl in der Magengegend  □ überhaupt nicht  □ gelegentlich  □ ziemlich oft  □ sehr oft |
| c) Mich überkommt eine ängstliche Vorahnung, dass etwas passieren könnte  □ ja, sehr stark  □ ja, aber nicht allzu stark  □ etwas, aber es macht mir keine Sorgen  □ überhaupt nicht | j) Ich habe das Interesse an meiner äußeren Erscheinung verloren  □ ja, stimmt genau  □ ich kümmere mich nicht so sehr darum, wie ich  sollte  □ möglicherweise kümmere ich mich zu wenig  darum  □ ich kümmere mich so viel darum wie immer |
| d) Ich kann lachen und die lustige Seite der Dinge sehen  □ ja, so viel wie immer  □ nicht mehr ganz so viel  □ inzwischen viel weniger  □ überhaupt nicht | k) Ich fühle mich rastlos, muss immer in Bewegung sein  □ ja, tatsächlich sehr  □ ziemlich  □ nicht sehr  □ überhaupt nicht |
| e) Mir gehen beunruhigende Gedanken durch den Kopf  □ einen Großteil der Zeit  □ verhältnismäßig oft  □ von Zeit zu Zeit, aber nicht allzu oft  □ nur gelegentlich/nie | l) Ich blicke mit Freude in die Zukunft  □ ja, sehr  □ eher weniger als früher  □ viel weniger als früher  □ kaum bis gar nicht |
| f) Ich fühle mich glücklich  □ überhaupt nicht  □ selten  □ manchmal  □ meistens | m) Mich überkommt plötzlich ein panikartiger Zustand  □ ja, tatsächlich sehr oft  □ ziemlich oft  □ nicht sehr oft  □ überhaupt nicht |
| g) Ich kann behaglich dasitzen und entspannen  □ ja, natürlich  □ gewöhnlich schon  □ nicht oft  □ überhaupt nicht | n) Ich kann mich an einem guten Buch, einer Radio- oder Fernsehsendung freuen  □ oft  □ manchmal  □ eher selten  □ sehr selten Page 10 |

**Explanation/Translation of questionnaire**

Page 1: Short description of aim of study and information about process of study.

Page 2: Declaration of consent

Page 3: Example on the handling of a numeric scale

Page 4:

Question 1: Pain scale.

Question 2: Alopecia

2 a: Detection of grade of alopecia according to CTCAE

2 b: Expectation of grade of alopecia after informed consent in comparison to actual extent of alopecia

2 c: Burden from alopecia, numeric scale

Question 3: Fatigue, construction of question see Question 2

Page 5 :

Question 4 : Nausea

4 a – c: Detection of grade of nausea according to CTCAE

4 d: Expectation of grade of nausea after informed consent in comparison to actual extent of nausea

4 e: Burden from nausea, numeric scale

Question 5: Vomiting

5 a – b: Detection of grade of vomiting according to CTCAE

5 c: Expectation of grade of vomiting after informed consent in comparison to actual extent of vomiting

5 d: Burden from vomiting, numeric scale

Page 6:

Question 6: Diarrhea

4 a – e: Detection of grade of diarrhea according to CTCAE

4 f: Expectation of grade of diarrhea after informed consent in comparison to actual extent of diarrhea

4 g: Burden from diarrhea, numeric scale

Page 7:

Question 7: Stomatitis

7 a – d: Detection of grade of stomatitis according to CTCAE

7 e: Expectation of grade of stomatitis after informed consent in comparison to actual extent of stomatitis

7 f: Burden from stomatitis, numeric scale

Question 8: Sensory neuropathy, construction of question see Question 5

Page 8:

Question 9: Skin toxicity, construction of question see Question 4

Question 10: Any other toxicity

10 a: Patients were encouraged to state any other toxicity

10 b: Interfering with daily life?

10 c: Life-threatening toxicity?

Page 9:

Question 11-1: If you were to decide, would you repeat chemotherapy with its effects and side effects?

Question 11-2: Patients were encouraged to fill in the blank: The minimum survival threshold I would undergo chemotherapy for again had to be ____.

Page 10: HADS
